# Effect of sacubitril/valsartan on cardiac remodeling compared with other renin–angiotensin system inhibitors: a difference-in-difference analysis of propensity-score matched samples

**DOI:** 10.1007/s00392-023-02306-0

**Published:** 2023-09-21

**Authors:** Erberto Carluccio, Frank L. Dini, Michele Correale, Giuseppe Dattilo, Michele Ciccarelli, Francesca Vannuccini, Stefano Sforna, Giuseppe Pacileo, Daniele Masarone, Laura Scelsi, Stefano Ghio, Carlo Gabriele Tocchetti, Valentina Mercurio, Natale Daniele Brunetti, Savina Nodari, Giuseppe Ambrosio, Alberto Palazzuoli, on behalf of the Working Group on Heart Failure of the Italian Society of Cardiology

**Affiliations:** 1 Cardiology and Cardiovascular Pathophysiology, S. Maria Della Misericordia Hospital, University of Perugia, Perugia, Italy; 2 Thoracic and Vascular Department, University of Pisa, Pisa, Italy; 3 Department of Cardiology, University Hospital Foggia, Foggia, Italy; 4 Department of Biomedical, Dental Sciences, and Morphofunctional Imaging, University of Messina, Messina, Italy; 5 Chair of Cardiology, Department of Medicine, Surgery and Dentistry, University of Salerno, Fisciano, Italy; 6 Department of Cardiology, University of Brescia and ASST Spedali Civili Di Brescia, Brescia, Italy; 7 Cardiovascular Diseases Unit, Cardio-Thoracic and Vascular Department, Le Scotte Hospital, University of Siena, Siena, Italy; 8 Heart Failure Unit, AORN Dei Colli, Monaldi Hospital, Naples, Italy; 9 Division of Cardiology, Fondazione I.R.C.C.S. Policlinico San Matteo, Pavia, Italy; 10 Department of Translational Medical Sciences, Federico II University, Naples, Italy; 11 CERICLET-Centro Ricerca Clinica E Traslazionale, University of Perugia, Perugia, Italy

**Keywords:** Sacubitril/valsartan, Neprilysin inhibitors, ARNI, Cardiac remodeling, Heart failure, RAS inhibitors

## Abstract

**Background:**

In patients with heart failure with reduced ejection fraction (HFrEF), treatment with sacubitril–valsartan (S/V) may reverse left ventricular remodeling (rLVR). Whether this effect is superior to that induced by other renin–angiotensin system (RAS) inhibitors is not well known.

**Methods:**

HFrEF patients treated with S/V (*n* = 795) were compared, by propensity score matching, with a historical cohort of 831 HFrEF patients (non-S/V group) treated with angiotensin-converting enzyme inhibitors or angiotensin receptor blockers (RAS inhibitors). All patients were also treated with beta-blockers and shared the same protocol with repeat echocardiogram 8–12 months after starting therapy. The difference-in-difference (DiD) analysis was used to evaluate the impact of S/V on CR indices between the two groups.

**Results:**

After propensity score matching, compared to non-S/V group (*n* = 354), S/V group (*n* = 354) showed a relative greater reduction in end-diastolic and end-systolic volume index (ESVI), and greater increase in ejection fraction (DiD estimator =  + 5.42 mL/m^2^, *P* = 0.0005; + 4.68 mL/m^2^, *P* = 0.0009, and + 1.76%, *P* = 0.002, respectively). Reverse LVR (reduction in ESVI ≥ 15% from baseline) was more prevalent in S/V than in non-S/V group (34% vs 26%, *P* = 0.017), while adverse LVR (aLVR, increase in ESVI at follow-up ≥ 15%) was more frequent in non-S/V than in S/V (16% vs 7%, *P* < 0.001). The beneficial effect of S/V on CR over other RAS inhibitors was appreciable across a wide range of patient’s age and baseline end-diastolic volume index, but it tended to attenuate in more dilated left ventricles (*P* for interaction = NS for both).

**Conclusion:**

In HFrEF patients treated with beta-blockers, sacubitril/valsartan is associated with a relative greater benefit in LV reverse remodeling indices than other RAS inhibitors.

**Graphical abstract:**

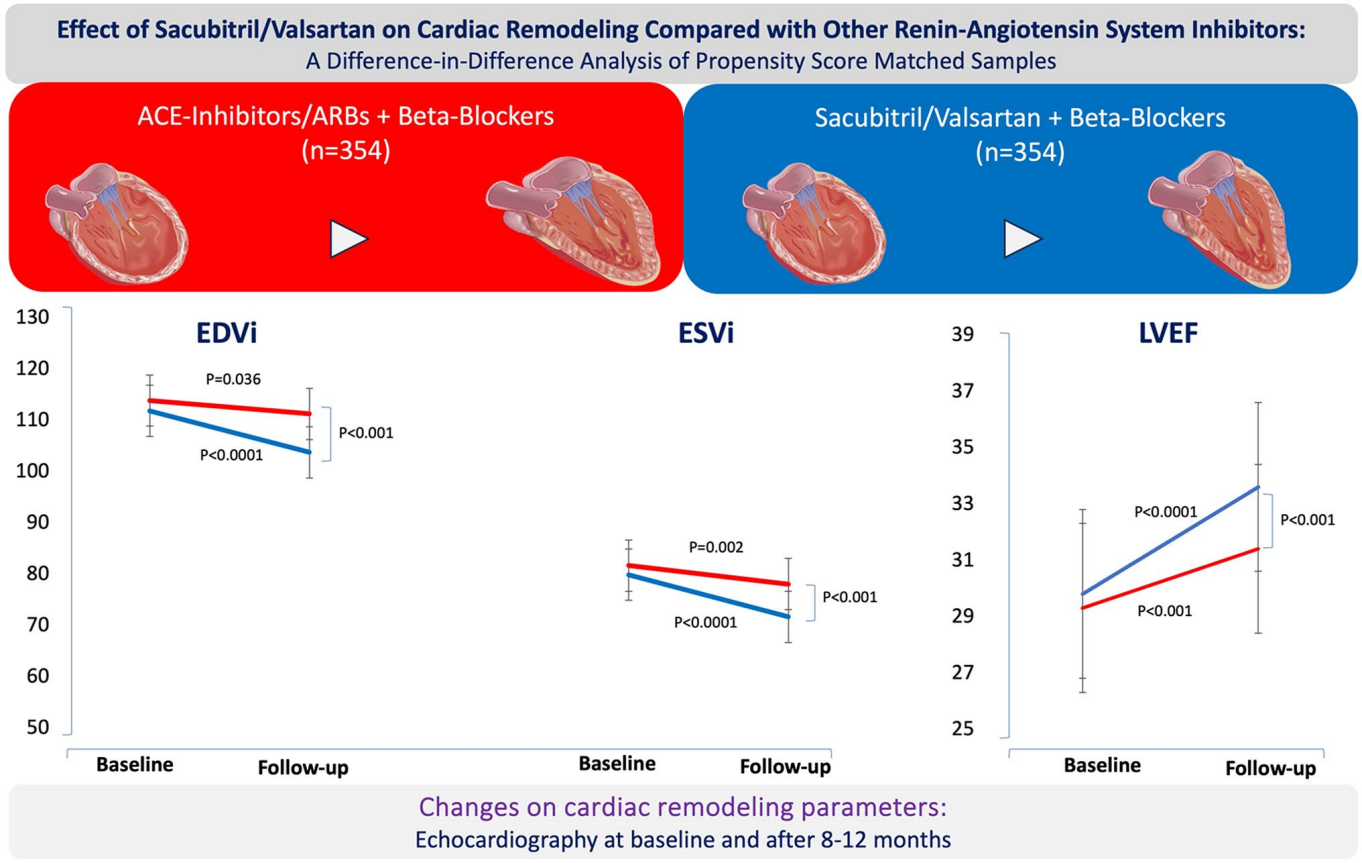

**Supplementary Information:**

The online version contains supplementary material available at 10.1007/s00392-023-02306-0.

## Introduction

Heart failure (HF) is the leading cause of morbidity and mortality, resulting in high health-care-related costs [1, 2]. As HF progresses, left ventricular (LV) end-diastolic volume (LVEDV) and end-systolic volume (LVESV) gradually increase, ventricular walls become thinner, leading to a more spherical left ventricle, and LV ejection fraction (LVEF) steadily decreases [3]. These alterations in LV architecture and chamber shape are labeled as adverse LV remodeling (aLVR) [3], and are an important determinant of the clinical process of HF being associated with adverse outcome [4]. On a histologic level, LVAR is associated with a combination of pathologic myocyte hypertrophy, apoptosis, myofibroblast proliferation, and interstitial fibrosis [3]. Although originally described after myocardial infarction, LVAR might develop in response to a variety of myocardial injuries and increased wall stress [5–7].

As opposed to aLVR, the term reverse LV remodeling (rLVR) refers to recovery from LV dilation and dysfunction that can be observed in a variable proportion of HF patients in response to guideline-recommended pharmacological or non-pharmacological (LV assist device, cardiac resynchronization therapy) HF therapies, and has been associated with improved outcome [4–6]. Indeed, neurohormonal antagonists [angiotensin-converting enzyme inhibitors (ACEi), beta-blockers, and angiotensin-receptor blockers (ARBs)] have all clearly demonstrated the capability of slowing disease progression and reversing LVR [4, 5, 8–10] which, at least in part, would mediate their favorable effects on prognosis. In a recent network meta-analysis, the association ACEi + beta-blockers was shown to be superior to ACEi alone in reducing LV volumes [11].

A recent breakthrough in the management of patients with HF with reduced EF (HFrEF) has been the introduction of sacubitril/valsartan (S/V), a combined ARB (valsartan)–neprilysin inhibitor (ARNI) drug. In the PARADIGM-HF (Prospective Comparison of ARNI with ACEi to Determine Impact on Global Mortality and Morbidity in Heart Failure) trial, S/V significantly decreased cardiovascular and all-cause mortality compared with the ACEi enalapril in patients with HFrEF [12]. These findings encouraged ARNI use in HFrEF patients [1, 2]. This novel class of drugs interferes with several key pathogenetic steps in HF progression, by means of powerful anti-remodeling and anti-fibrotic effects. Indeed, several trials provided evidence that S/V can promote rLVR with an increase in LVEF, and a significant reduction of LV volumes [13–15]. However, whether the salutary effect of S/V on rLVR is superior to that induced by other renin–angiotensin system (RAS) inhibitors (ACEi/ARBs) is not well known. To test this hypothesis, in the current study patients who started S/V in a multicenter Italian registry were compared, using propensity score-matching procedure, with an HFrEF cohort not on S/V but treated with other RAS inhibitors. All patients in both cohorts were also treated with beta-blockers.

## Methods

### Study populations

**Fig. 1:**
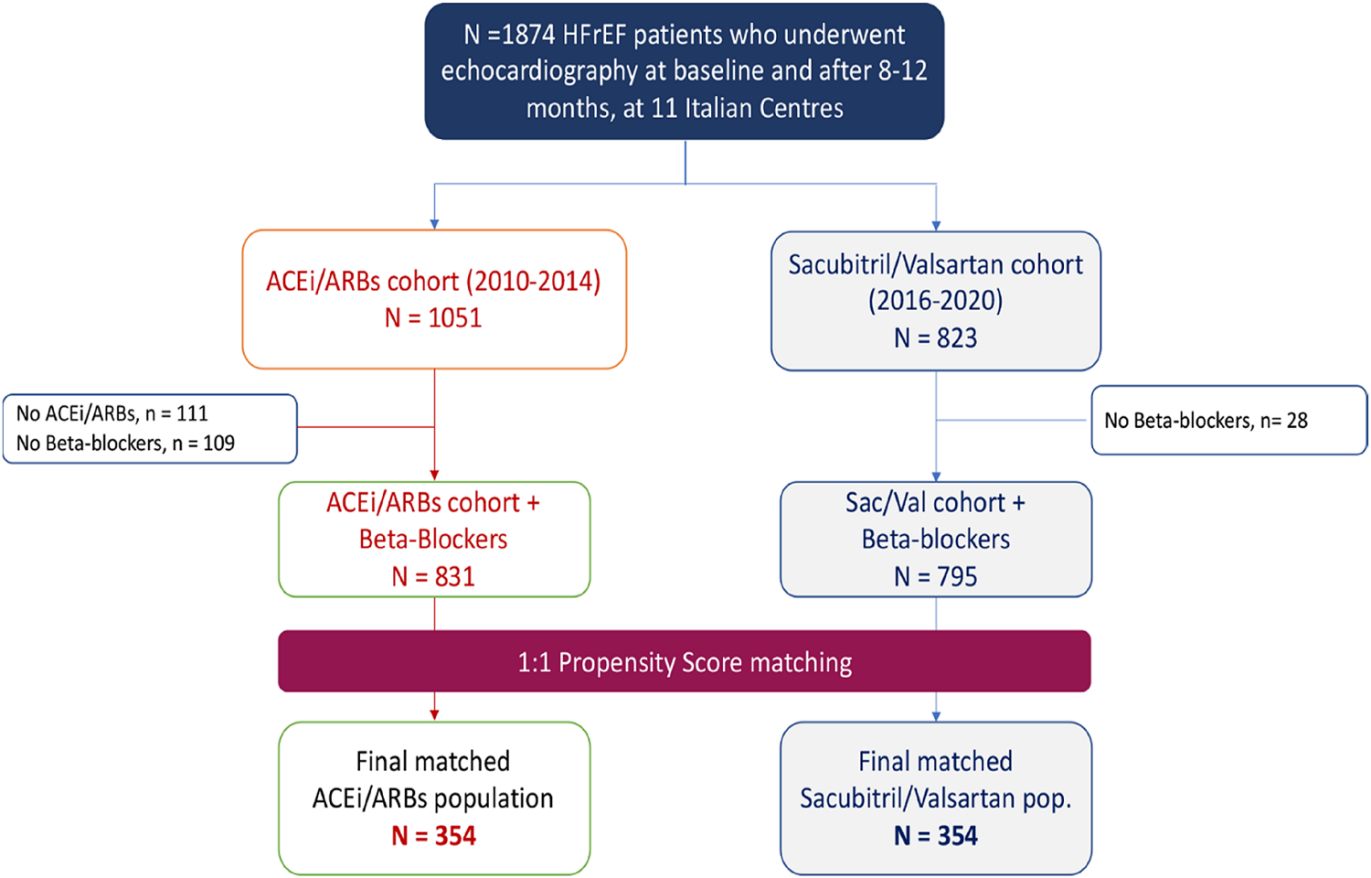
Study population

The S/V cohort consists of ambulatory HFrEF patients with optimized standard-of-care therapy for chronic HFrEF and indication for initiating S/V therapy, who were prospectively included in a multicenter, open-label registry from 11 Italian academic hospitals before starting S/V therapy (NCT04397302) from December 2016 to October 2020 [16, 17]. Per protocol, patients underwent echocardiography at baseline and 8–12 months after treatment. Detailed information on study protocol, patients’ medical history, including medications and laboratory data, as well as inclusion and exclusion criteria have been described elsewhere [16, 17] (see also *Supplemental Material Data* file). Briefly, for eligible patients on ACE Inhibitors, S/V was started after a washout period of 36-h at a preferential dose of 49/51 mg b.i.d., or 24/26 mg b.i.d. for those taking a low dose of ACE inhibitors. At each participating site, S/V dose was tentatively doubled every 2–3 weeks to reach the target maintenance dose of 97/103 mg b.i.d., except in patients with systolic blood pressure (BP) less than 100 mmHg or who developed drug-related adverse events [symptomatic hypotension, hyperkalaemia > 5.5 mEq/l, or a decrease in estimated glomerular filtration rate (eGFR) to < 60 ml/min]. Only patients who were also taking beta-blockers were eligible for this study (Fig. 1).

Non-S/V cohort consists of a retrospective evaluation of a cohort of 831 chronic HFrEF outpatients evaluated in 7 Italian Hospitals between January 2010 and December 2014 (before the introduction of S/V), who also underwent a second complete echocardiographic examinations 8–12 months afterwards (Fig. 1) [18] (see also *Supplemental Material Data* file). Only patients treated with ACE inhibitors/ARBs and beta-blockers were considered for this study.

In both cohorts, demographic, clinical, laboratory, and echocardiographic data were collected at study entry; clinical and echocardiographic data were re-evaluated at the reassessment. The study was conducted according to institutional guidelines, national legal requirements, European standards, and the revised Declaration of Helsinki. All patients provided written informed consent for anonymous collection and publication of their clinical data.

### Echocardiography

Both at baseline and follow-up reassessment, LVEDV, LVESV, and LVEF were calculated according to the biplane Simpson’s method according to the recommendations of the American Society of Echocardiography and European Association of Cardiovascular Imaging [19]. Chamber volume measures were indexed to body surface area [19]. Significant reverse LVR (rLVR) was defined as ≥ 15% reduction in LVESV index (LVESVI) at follow-up re-assessment [19], while adverse LVR (aLVR) was defined as ≥ 15% increase in LVESVI at follow-up. Patients in whom LVESVI showed less than 15% increase or decrease were defined as “unchanged”.

Doppler examinations included assessment of early diastolic filling velocity (E wave) and early diastolic mitral annular velocity (e’) at the septal and lateral side of mitral annulus; an averaged E/e’ ratio > 14 was considered a surrogate marker of increased LVFP [20]. Mitral regurgitation severity was assessed using color, continuous-wave Doppler, as well as conventional quantitative parameters according to European Society of Echocardiography recommendations. Patients were then categorized as severe and non-severe mitral regurgitation. All measurements were repeated at least three times and the average value was calculated.

### Statistical analysis

If normally distributed, continuous data are presented as mean ± standard deviation (SD) and compared (at each time point) by t *test* for independent samples. In case of skewed distribution, median and interquartile range (IQR: Q1–Q3) are shown and Kruskal–Wallis test used for comparison. Categorical variables are reported as *n* and percentage and compared by chi-squared test. To compare differences between baseline and follow-up evaluation in each treatment group, a paired-sample *t* test was used for normally distributed variables and a Wilcoxon matched-pairs signed-rank test for variables with skewed distribution. NT-proBNP was logarithmically transformed for statistical analyses.

Propensity score (PS) matching was used to reduce the effect of confounders caused by differences in baseline demographic, clinical, echocardiographic, and laboratory characteristics between sacubitril/valsartan and other RAS inhibitor groups (package “*MatchIt”* in R). PS was obtained by non-parsimonious multivariable logistic regression, with treatment strategy (sacubitril/valsartan vs RAS inhibitors group) as dependent variable, and NYHA class, body mass index, mean blood pressure, baseline LV end-diastolic, end-systolic volumes and EF, baseline E/e’ ratio, severe MR (yes/no), atrial fibrillation (yes/no), diabetes (yes/no), hypertension (yes/no), previous CRT and/or ICD implantation, and LogNT-proBNP, as independent variables. To create paired samples of patients with similar PS, one-to-one “*nearest neighbour*” matching was used, with caliper = 0.1 and without replacement. The procedure yielded 354 well-matched pairs. The success of the PS matching was assessed by checking standardized differences between groups before and after matching [21]. Balancing was considered successful if the standardized differences were < 10%.

To estimate the effect of sacubitril/valsartan on cardiac remodeling among HFrEF patients, the difference-in-difference (DID) estimator analysis was adopted [22]. The DID estimator indicated whether patients treated with sacubitril/valsartan had more cardiac functional and structural changes over time than patients treated with other RAS inhibitors.

To assess whether the effect of S/V versus other RAS inhibitors differed across the spectrum of age and LV dimension at baseline, the interaction between age (as continuous variable) and treatment, and LVEDVI and treatment on the occurrence of aLVR was tested in a logistic regression model. A fractional polynomial was constructed for both age and LVEDVI and entered the model as interaction terms with treatment [23]. Results of the interaction were displayed graphically using the “*mfpi*” command in STATA [23]. Statistical analyses were performed with STATA-17 (StataCorpMP), and R statistic (version 4.2.2). A two-sided p value < 0.05 was considered statistically significant.

## Results

Before matching, out of 1626 total HFrEF patients who were included in this study, 795 were in the S/V group, and 831 were in the non-S/V group (Fig. 1). The baseline characteristics of the participants are summarized in Supplemental Table-S1. Seventeen variables (Table-S1 and Figure-S2 in *Supplemental material data file*) resulted associated with treatment group and defined the PS for each patient. Using the PS, S/V and non-S/V cohorts were then matched, providing 354 pairs of patients, which defined the investigational cohort of this analysis. After matching, patients treated with S/V and non-S/V group were balanced across all but four baseline variables (gender, heart rate, CKD, and ischemic etiology; Table 1). Optimally matched patients had similar final propensity scores (Figure-S2 in *supplemental material file*); the standardized differences for the confounders were all bigger than 0.1 before PS matching (Supplemental Figure-S2), but smaller than 0.1 after matching, indicating negligible imbalance among treatment groups.

**Table 1:** Clinical and echocardiographic characteristics of the matched population

Variables	Total (708)	ACEi/ARBs (*n* = 354)	Sac./Valsartan (*n* = 354)	*P* value
Age	63.9 ± 11.5	64.6 ± 11.2	63.1 ± 11.8	0.082
Males	588 (83.1%)	282 (79.6%)	306 (86.4%)	0.016
BMI	26.9 ± 4.5	26.9 ± 4.1	26.9 ± 4.9	0.965
Systolic BP	117.8 ± 15.2	117.1 ± 15.3	118.4 ± 15.0	0.245
Diastolic BP	72.7 ± 9.7	73.1 ± 9.7	72.3 ± 9.6	0.252
Heart rate (bpm)	69.2 ± 11.1	70.5 ± 11.1	67.9 ± 11.0	0.002
NYHA class > 2	206 (29.1%)	104 (29.4%)	102 (28.8%)	0.869
Diabetes	173 (24.4%)	87 (24.6%)	86 (24.3%)	0.930
CKD	256 (36.2%)	146 (41.2%)	110 (31.1%)	0.005
Hypertension	331 (46.8%)	169 (47.7%)	162 (45.8%)	0.598
Ischemic etiology	363 (51.3%)	203 (57.3%)	160 (45.2%)	0.001
Atrial fibrillation	110 (15.5%)	51 (14.4%)	59 (16.7%)	0.407
NT-proBNP	609 (276–1161)	606 (276–1088)	609 (276–1167)	0.692
Therapy				
Loop diuretics	522 (73.7%)	235 (66.4%)	240 (67.8%)	0.345
Aldosterone antagonists	468 (66.1%)	224 (63.3%)	244 (68.9%)	0.112
Beta-blockers	708 (100%)	354 (100%)	354 (100%)	–
CRT	244 (34.5%)	117 (33.1%)	127 (35.9%)	0.429
ICD	374 (52.8%)	191 (54.0%)	183 (51.7%)	0.547
Echocardiography				
EDVI (mL/m2)	112.8 ± 38.6	113.9 ± 37.1	111.8 ± 40.1	0.478
ESVI (mL/m2)	80.7 ± 31.8	81.6 ± 29.7	79.8 ± 33.7	0.466
LV ejection fraction (%)	29.5 ± 6.5	29.3 ± 6.1	29.8 ± 6.9	0.310
E/e’ ratio	14.7 ± 7.2	14.8 ± 7.4	14.6 ± 7.0	0.726
Severe MR	217 (30.6%)	112 (31.6%)	105 (29.7%)	0.568

*P* values by *t* test for continuous variables and chi-squared test for binary/categorical variables

### Follow-up comparisons

Time to follow-up echocardiographic reassessment was comparable between S/V and non-S/V group (9 ± 2 months vs 8.9 ± 1.8, *P* = 0.4846). The impact of S/V over other RAS inhibitors on LVCR is reported in Table 2. In both treatment groups, LVEDVI (S/V, P < 0.0001; non-S/V, P = 0.0360), as well as LVESVI (S/V, P < 0.0001; non-S/V, P = 0.0017) significantly reduced at follow-up, and LVEF increased (P < 0.0001 for both groups). However, at follow-up reassessment both LVEDVI (*P* = 0.0158) and LVESVI (*P* = 0.0133) were significantly lower, and LVEF higher (P = 0.0001), in S/V compared to non-S/V group (Table 2 and Graphic Abstract). Therefore, in comparison with non-S/V group both LVEDVI and LVESVI had more relative decrease among S/V patients, with a negative significant DID estimator (-5.42 and -4.68 mL/m^2^, respectively, P < 0.001 for both), while LVEF had more relative increase with a positive significant DID estimator (+ 1.76%, P = 0.002, Table 2). Interestingly, the reduction in LVESVI by S/V compared to other RASi was already significant at low-dose of the drug (-8.2 ± 21 ml/m^2^ versus -3.7 ± 25 mL/m^2^, *P* = 0.0314), and the beneficial effect seems to increase increased dosage of S/V (P = 0.0006 for trend, Figure-S3 in *Supplemental Material data file*).

**Table 2:** Changes on cardiac remodeling parameters evaluated by difference-in-difference (DID) analysis

Parameters	S/V group (*n* = 354)		Non-S/V group (*n* = 354)		DID estimator	95% CI	*P*
	Baseline	Follow-up	Baseline	Follow-up			
EDVi [mL/m^2^]	111.8 ± 40.1	103.7 ± 41.5	113.8 ± 37.1	111.2 ± 40.7	– 5.42	– 8.45; – 2.38	< 0.001
ESVI [mL/m^2^]	79.8 ± 33.7	71.6 ± 34.2	81.6 ± 29.7	78.0 ± 34.6	– 4.68	– 7.43; – 1.93	< 0.001
LVEF [%]	29.8 ± 6.9	33.6 ± 8.8	29.3 ± 6.1	31.4 ± 9.5	+ 1.76	– 0.65; 2.88	0.002

**Fig. 2:**
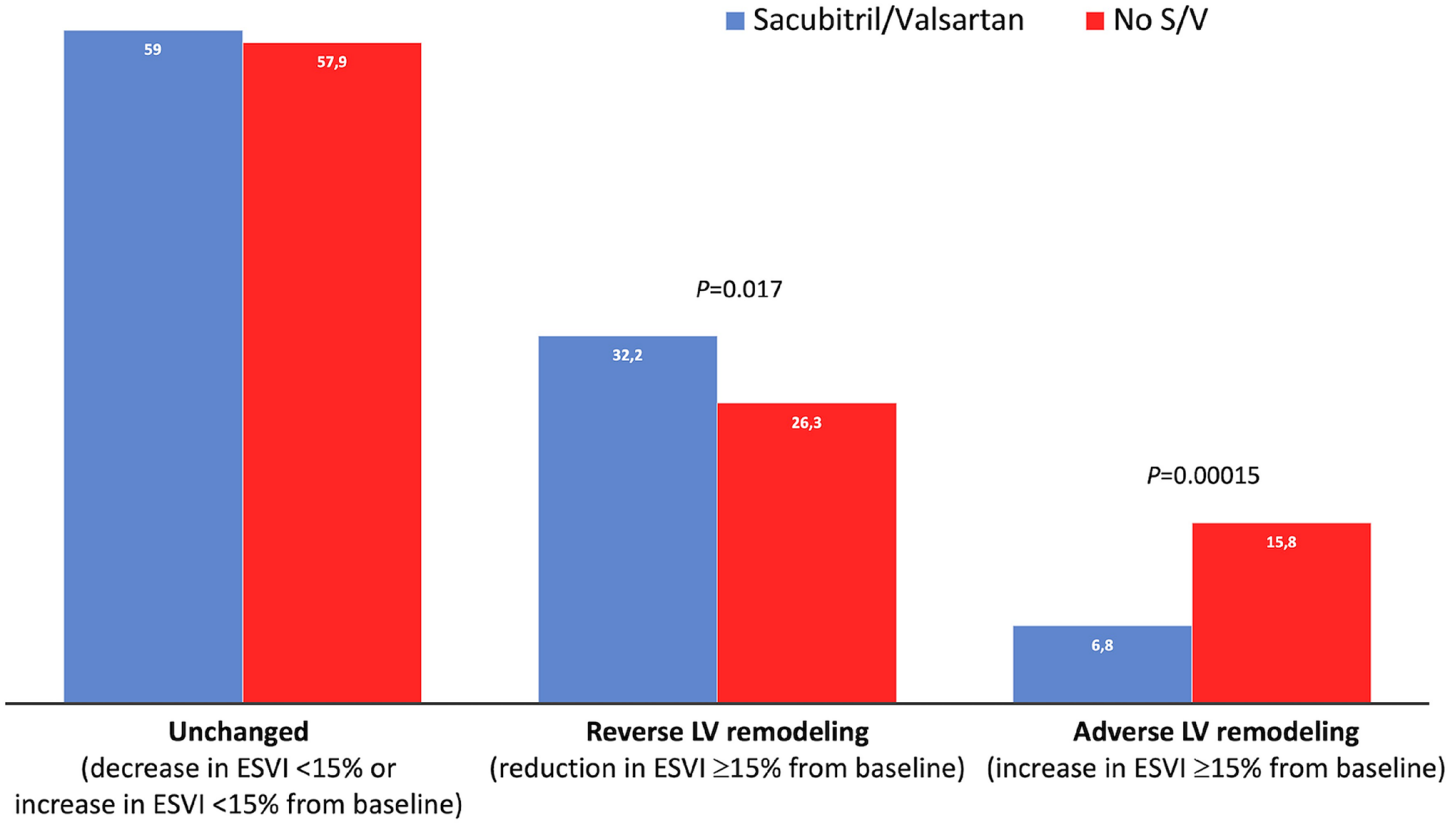
Prevalence of LV reverse remodeling and adverse cardiac remodeling (A-LVR) according to treatment groups

A rLVR (≥ 15% reduction in LVESVI at reassessment) was found in 214 (30%) patients, while an aLVR (≥ 15% increase in LVESVI at follow-up) was found in only 80 (11%) patients of the total population. A rLVR was more prevalent among patients treated with S/V than with other RASi (34% vs 26%, P = 0.017), while an aLVR showed an opposite distribution being more prevalent in non-S/V group than in patients treated with S/V (16% vs 7%, P < 0.001; Fig. 2).

**Fig. 3:**
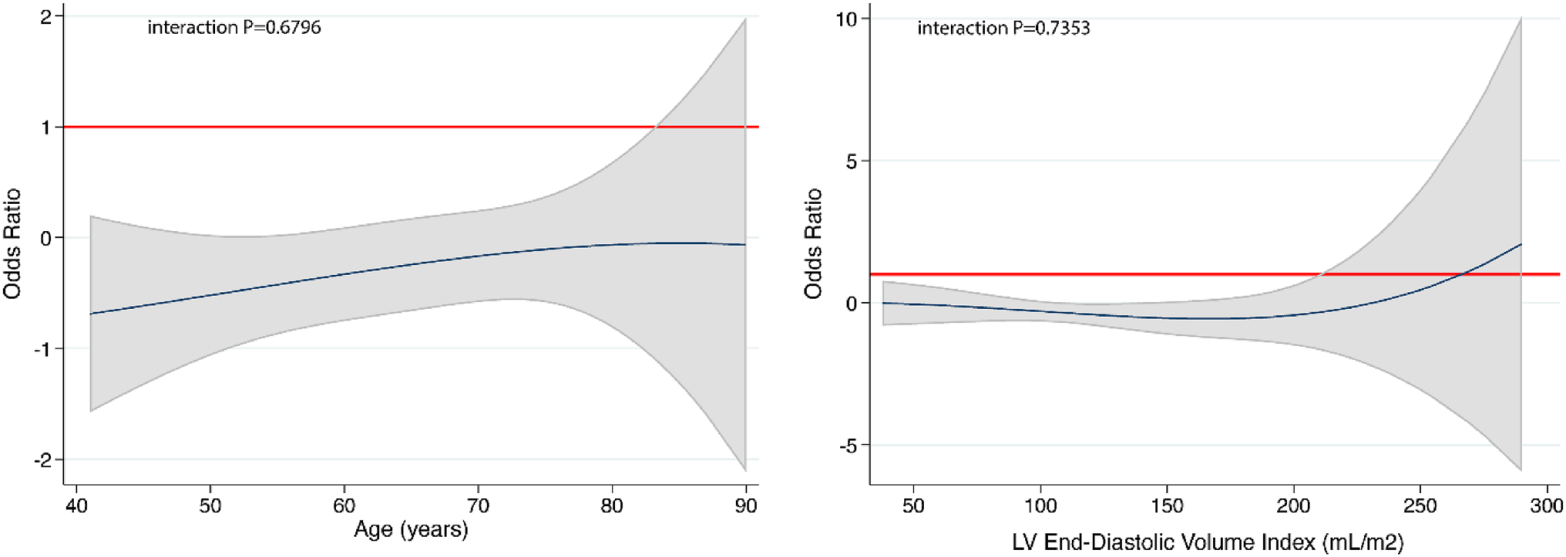
Sacubitril/valsartan to other RAS inhibitors odds ratio (line) and 95% confidence intervals (shaded area) for adverse LV cardiac remodeling (LVCR) according to age (left panel) and baseline LV end-diastolic volume index (right panel). An odds ratio of 1.0 is indicated by the solid horizontal line. An odds ratio of < 1.0 favours sacubitril/valsartan

Figure 3 graphically shows the effect of S/V compared with other RAS inhibitors relatively to aLVR using fractional polynomial analysis. These graphs show the odds ratio for S/V vs. Other RAS inhibitors, adjusted for chronic kidney disease and ischemic etiology, at each age (left panel) or baseline LVEDVI (right panel), i.e. with these variables treated as a continuous variables. Consistent with the categorical analysis, risk of an aLVR in the S/V group was lower than in the non-S/V group across the age spectrum, even in the most elderly patients (interaction *P* = 0.6796). Similarly, the risk of aLVR was also lower in S/V group across a wide range of LVEDVI and showed a flat curve indicating that the magnitude of the effect of S/V on LVCR was similar across the spectrum of LVEDVI (interaction *P* = 0.7353), except for the most dilated left ventricles where the 95% confidence intervals became wide, suggesting an attenuation of the beneficial effect of S/V in more advanced cardiac remodeling.

## Discussion

The present study showed that patients with HFrEF had a relative greater reduction in LV volumes and a greater increase in LVEF when treated with S/V + beta-blockers than when treated with ACE inhibitors/ARBs + beta-blockers. The amount of the reduction in LVESVI increased with increasing dosage of S/V. When defined categorically, a reverse LV remodeling was more prevalent, while adverse LV remodeling less prevalent among S/V patients compared to non-S/V patients. The additive beneficial effect of S/V on LV remodeling was consistent across a wide range of age and seems to attenuate in the presence of more dilated left ventricles.

Over the past decades, advances in pharmacological therapies provided several breakthroughs in the knowledge of rLVR in HFrEF patients, especially on its impact in reducing HF-hospitalization and mortality. Indeed, ACE inhibitors/ARBs, ARNI, Beta-blockers, and mineralocorticoid receptor antagonists (MRAs) are now widely accepted and guideline-recommended therapeutic approaches in reducing morbidity and mortality in HFrEF [1, 2]. It has been argued that the beneficial effect on outcome of these guideline-recommended therapies might be mediated, at least in part, by their positive effect on rLVR [3].

Indeed, in 2010, Kramer DG et al. through a meta-analytic approach, evaluated 30 mortality trials of 25 drug/device therapies and 88 remodeling trials of the same therapies. They demonstrated a significant association between short-term therapeutic effects of a drug on parameters of LV remodeling and longer-term therapeutic effects on mortality in patients with LV dysfunction [4], suggesting that drug effects on LV remodeling should be viewed as suggestive of the intervention’s potential effect on mortality. In the last years, the incremental use of combinations of disease-modifying therapies (ACEIs, ARBs, Beta-blockers, MRAs, and ARNI) has resulted in a progressive improvement in mortality and hospitalization outcomes in HFrEF [24], and the combinations including the most recently developed drugs, such as sacubitril/valsartan (ARNI + beta-blockers + MRA), appeared the most efficacious among the different possible combinations [24]. Accordingly, recent meta-analyses found that combination therapies exert more benefits on rLVR for patients with HFrEF [11, 25]. Bao J et al. reported that among all possible combinations, ARNI + Beta-blockers, and ARNI + Beta-blockers + MRA emerged as the top two effective dual and triple combinations in LVEF improvement, respectively [11]. In addition, the so-called new “*Golden Triangle*” of ARNI + Beta-blockers + MRA was shown to be superior to the combinations based on ACEI + Beta-blockers + MRA or ARBs + Beta-blockers + MRA in LVEF improvement [11]. Wang Y et al. in their meta-analysis, that compared the effects of ARNI versus ACE inhibitors or ARBs on LVRR indices, analyzed twenty studies including a total 10.175 patients [25]. They found that ARNI distinctly improved LV size and hypertrophy compared with ACE inhibitors/ARBs in HFrEF patients, even after short-term follow-up [25]. Both these meta-analyses, however, have some limitations: some analyzed studies were conference abstracts with unrefined design methodologies, which could have affected the overall study quality [25]; furthermore, the number of studies on some comparisons was limited, and the follow-up reassessment lasted from few weeks to several months, which may have influenced the process of cardiac remodeling in HFrEF patients. Therefore, the results should be interpreted with caution.

Prospective data regarding S/V and cardiac remodeling are limited. In a prospective, randomized trial enrolling patients with asymptomatic LV systolic dysfunction late after myocardial infarction, treatment with S/V did not show a significant reverse remodeling effect compared with valsartan [26]. However, patients enrolled in this trial were asymptomatic for their LV systolic dysfunction with very low levels of NT-proBNP suggesting a low degree of neurohormonal activation [26]. The PROVE-HF study reported a significant correlation between the degree of change in LVESVI and the change in NT-proBNP from baseline to follow-up, but this study lack of a control group [14] and was not able to demonstrate a superior effect of ARNI over other RAS inhibitors. The EVALUATE-HF trial randomized 464 HFrEF patients to S/V versus enalapril [13]. Although this trial failed to demonstrate a significant effect of S/V on the primary endpoint (central aortic stiffness), significant reductions were seen with S/V in selected secondary echocardiographic endpoints, including LVEDV and LVESV, suggesting improvement in cardiac remodeling. However, no difference was noted in LVEF and other systolic functional parameters between treated groups [13], and only 85% of patients in this trial were also treated with beta-blockers and only 25% received a MRAs agent, which could have affected the results.

In the present study performed in a real-world setting, we used PS to match two distinct HFrEF populations that shared the same protocol having a repeated echocardiogram 8–12 months after starting HF therapies. This allowed us to balance the treatment groups on confounding factors to make them comparable so that we can draw conclusions about the causal impact of S/V on LVRR using observational data. We found a relative greater reduction in LV volumes with S/V than with ACE inhibitors/ARBs in HFrEF also treated with beta-blockers. Interestingly, although the amount of this beneficial effect of S/V on LVRR seems to increase with increasing dosage of the drug, the effect induced by S/V on LVRR indices was notable also in HFrEF patients who failed to reach the target dose (Figure-S2 in *supplemental material* data).

We also found that the benefit of S/V over ACE inhibitors/ARBs is attenuated in very large LV volumes but persists in small LVs. This finding provides an opportunity to hypothesize a probable course of progression and regression of LV remodeling. According to this view, the early stage of the disease, when LV remodeling is only minimal, may reverse to normal, and thus this may also be the “golden time” for drug implementation and dosage optimization. Over time, LV remodeling progressively increases, also accompanied by irreversible changes to LV structure, and the likelihood of benefit of reverse remodeling may decline, blunting the effect of therapy [6, 7]. These considerations might support a possible role of ARNI for the management of patients with HF with mildly reduced EF (HFmrEF). The amount of LV remodeling and functional improvement seen in our study after S/V initiation is consistent with that of other prospective observational studies [27, 28] and highlights the need for serial echocardiographic reassessment in HFrEF after diseased-modifying HF therapies that could help saving the indication for implantable cardioverter-defibrillator for arrhythmic primary prevention [28], according to the current criteria.

### Limitations of the study

The main limitation of our study is the retrospective nature. However, it is a multicenter study involving several long-standing experienced centers on the treatment of HF in Italy. We did not use a core-lab for blinded assessment/adjudication of the echocardiograms. However, each participating center had great experience in the imaging assessment of HF patients and the expertise of this group of investigators has already been extensively put to use in a research network [16, 17]. Furthermore, we used the PS matching analysis to overcome the absence of a randomized design. This type of testing reduces bias when comparing treatment strategies in non-randomized studies [21] by matching patients on the basis of many confounders simultaneously prior to analysis. However, unlike randomized controlled trials in which balancing is based both on observed and unobserved characteristics, PS matching leads to balanced patient groups only for recorded covariates. Thus, PS analyses have the limitation that unmeasured confounders may still be present. To further strengthen the analysis, we used the DiD methodology [22]. DiD is a quasi-experimental design that makes use of longitudinal data from treatment and control groups to obtain an appropriate counterfactual information to estimate a causal effect. This analysis is a useful technique when randomization on the individual level is not possible, as in observational settings where exchangeability cannot be assumed between the treatment and control groups. The approach removes biases in post-intervention period comparisons between the treatment and control group that could be the result of permanent differences between those groups, as well as biases from comparisons over time in the treatment group that could be the result of trends due to other causes of the outcome. The main limitation of this kind of analysis is that it cannot be used if comparison groups have different outcome trend [22], but this is not the case in the present study. Finally, we did not have information about the maximum RAS inhibitors dosage reached in the non-S/V group. Therefore, we cannot exclude that also in this group the effect on LV remodeling was dose-dependent. However, even in this group, the follow-up re-evaluation was performed after optimization of HF drugs, and we can assume that patients were in maximum tolerated dose of RAS inhibitors at the time of echocardiographic reassessment. Our data come out from pre-SGLT2 inhibitors introduction. Future prospective studies may evaluate the impact of additional therapy on LV chamber and function with combined SV/SGLT2 treatment.

### Conclusions

In HFrEF patients treated with beta-blockers, sacubitril/valsartan is associated with a relative greater benefit in LV reverse remodeling indices and low prevalence of ongoing LV remodeling compared to angiotensin-converting-enzyme inhibitors or angiotensin-receptor blockers alone. The beneficial effect of S/V on rLVR over other RAS inhibitors was appreciable across a wide range of patient’s age and seems to attenuate in the presence of more enlarged left ventricles.

### Supplementary Information

Below is the link to the electronic supplementary material.

Supplementary file1 (DOCX 3702 KB)
