# Supplementary material for: Effect of sacubitril/valsartan on cardiac remodeling compared with other renin–angiotensin system inhibitors: a difference-in-difference analysis of propensity-score matched samples
Source: Clin Res Cardiol. 2023 Sep 21;113(6):856–65. doi: 10.1007/s00392-023-02306-0 (PMC11108945; doi:10.1007/s00392-023-02306-0)
Supplement: Supplementary file 1 — Supplementary file1 (DOCX 3702 KB) [file 392_2023_2306_MOESM1_ESM.docx]

Effect of Sacubitril/Valsartan on Cardiac Remodeling Compared with other Renin-Angiotensin System inhibitors:

A difference-in-difference analysis of propensity score matched samples

**Supplemental Marterial**

**Cohort Sacubitril/Valsartan: Inclusion and Exclusion Criteria**

*Entry criteria* were: LVEF ≤40%within the preceding 6 months (according to local measurement), clinical stability on a stable dose of loop diuretic since at least 2 weeks preceding study start, optimized medical therapy (defined as treatment for ≥6 months on maximum tolerated dose of an ACE-Inhibitor or angiotensin receptor antagonists [ARBs] and a betablocker, possibly in association with a mineralcorticoid-receptor antagonist [MRA]), suitability for on-label S/V treatment as per standard of care, and availability to undergo repeat echocardiographic examinations 8–12 months afterwards.

*Exclusion criteria* were: history of hypersensitivity/allergy, or suspected contraindication, to ACE-inhibitors/angiotensin receptor blockers, congenital heart disease, severe valvular disease, restrictive physiology, valvular surgery, recent (within the previous three months) acute coronary syndromes revascularization or cardiac resynchronization therapy (CRT), scheduled revascularization or CRT, history of stroke, and poor acoustic windows.

Patients lost prior to follow-up echocardiographic evaluation, or who underwent cardiac surgery, coronary or mitral interventions before follow-up re-evaluation, were excluded from final analysis, as were patients with incomplete/missing follow-up data.

**Non-Sacubitril/Valsartan Cohort: Inclusion and Exclusion Criteria**

*Inclusion criteria* were: LV ejection fraction (LVEF)<40%; etiology caused by coronary artery

Disease (diagnosed on the basis of documented previous myocardial infarction or significant disease on coronary arteriography) or hypertensive heart disease or idiopathic cardiomyopathy; stable clinical conditions over the last 3 months; age > 18 years. HF was defined by cardiologist-adjudicated HF diagnosis according to the Framingham criteria. Inclusion criteria were

assessed at the first echocardiographic evaluation.

*Exclusion criteria* were: myocardial infarction, or coronary artery bypass graft, or percutaneous coronary angioplasty in the previous 3 months; implantation of a cardiac resynchronization device in the previous 3 months; organic valvular heart disease; history of pulmonary embolism and any life-threatening condition with adverse prognosis other than

cardiovascular disease; and any lung disease causing precapillary pulmonary hypertension. scheduled revascularization or CRT. Acute ischemic cardiac events and revascularization procedures during the time interval between the two echo examinations were also considered exclusion criteria.

**The difference-in-difference (DID) estimator**

The difference-in-difference (DID) estimator analysis focused on comparing cardiac functional and structural changes that occurred between baseline and follow-up in both intervention (sacubitril/valsartan) and control (other-RAS-inhibitors) groups. The model that was used for this DID regression is written as: Y*_it_* = β_0_ + β_1_G_i_ + β_2_T_t_+β3G_i_T_t_, where, Y_it_ is the parameter Y in patient *i* measured at time *t* (baseline or follow-up); β_0_ is a constant; G_i_ is a dummy that indicates whether the patient was in the sacubitril/valsartan group (G_i_ = 1) or in the other-RASi group (G_i_ = 0); T_t_ is a dummy that indicates whether the parameter was measured at the follow-up (T_t_ = 1) or at the baseline (T_t_ = 0). The main parameter of interest was β_3_ (the DID estimator), which indicated whether patients treated with sacubitril/valsartan had more cardiac functional and structural changes over time than patients treated with other-RAS inhibitors.

| **Supplemental Table-S1: Clinical and Echocardiographic characteristics of the total population** | | | | | |
| --- | --- | --- | --- | --- | --- |
| Variables | **Total**  (1626) | **ACEi/ARBs**  (n=831) | **Sac./Valsartan**  (n=795) | P-value |  |
| Age, years | 64.0 ± 11.9 | 63.6 ± 12.0 | 64.3 ± 11.8 | 0.264 |  |
| Males | 1351 (83.1%) | 683 (82.2%) | 668 (84.0%) | 0.324 |  |
| BMI | 27.2 ± 4.9 | 26.8 ± 4.2 | 27.7 ± 5.5 | 0.0001 |  |
| Systolic BP, mmHg | 119.1 ± 16.2 | 116.9 ± 16.3 | 121.4 ± 15.8 | <0.0001 |  |
| Diastolic BP, mmHg | 72.5 ± 10.0 | 71.3 ± 10.2 | 73.7 ± 9.6 | <0.0001 |  |
| Mean BP, mmHg | 88.0 ± 10.8 | 86.5 ± 11.0 | 89.6 ± 10.4 | <0.0001 |  |
| Heart Rate, bmp | 69.7 ± 11.6 | 70.1 ± 11.4 | 69.2 ± 11.9 | 0.114 |  |
| NYHA class >2 | 483 (29.7%) | 230 (27.7%) | 253 (31.2%) | 0.067 |  |
| Diabetes | 447 (27.5%) | 193 (23.2%) | 254 (31.9%) | <0.0001 |  |
| CKD | 545 (33.5%) | 273 (32.9%) | 272 (34.2%) | 0.561 |  |
| Hypertension | 826 (50.8%) | 348 (41.9%) | 478 (60.1%) | <0.0001 |  |
| Ischemic etiology | 813 (50.0%) | 418 (50.3%) | 395 (49.7%) | 0.804 |  |
| Atrial Fibrillation | 255 (15.7%) | 96 (11.6%) | 159 (20.0%) | <0.0001 |  |
| NT-proBNP | 666 (242-1426) | 380 (126-806) | 1014 (534-2524) | 0.0001 |  |
| ***Therapy*** |  |  |  |  |  |
| Loop diuretics | 1299 (79.9%) | 609 (73.3%) | 690 (86.8%) | <0.0001 |  |
| Aldosterone Antagonists | 1078 (66.3%) | 527 (63.4%) | 551 (69.3%) | 0.012 |  |
| Beta-Blockers | 1626 (100%) | 831 (100%) | 795 (100%) | 0.999 |  |
| CRT | 571 (35.1%) | 365 (43.9%) | 206 (25.9%) | <0.0001 |  |
| ICD | 859 (52.8%) | 353 (42.5%) | 506 (63.7%) | <0.0001 |  |
| ***Echocardiography*** |  |  |  |  |  |
| EDVI | 112.9 ± 38.8 | 120.4 ± 41.5 | 105.2 ± 34.1 | <0.0001 |  |
| ESVI | 81.2 ± 32.3 | 87.5 ± 34.4 | 74.6 ± 28.4 | <0.0001 |  |
| LV Ejection Fraction | 29.2 ± 6.5 | 28.3 ± 6.4 | 30.1 ± 6.5 | <0.0001 |  |
| E/e’ ratio | 14.9 ± 7.3 | 15.5 ± 8.1 | 14.2 ± 6.1 | <0.0001 |  |
| Severe MR | 538 (33.1%) | 236 (28.4%) | 302 (38.0%) | <0.0001 |  |
| P-values by t-test for continuous variables and Chi2 test for binary/categorical variables. | | | | | |


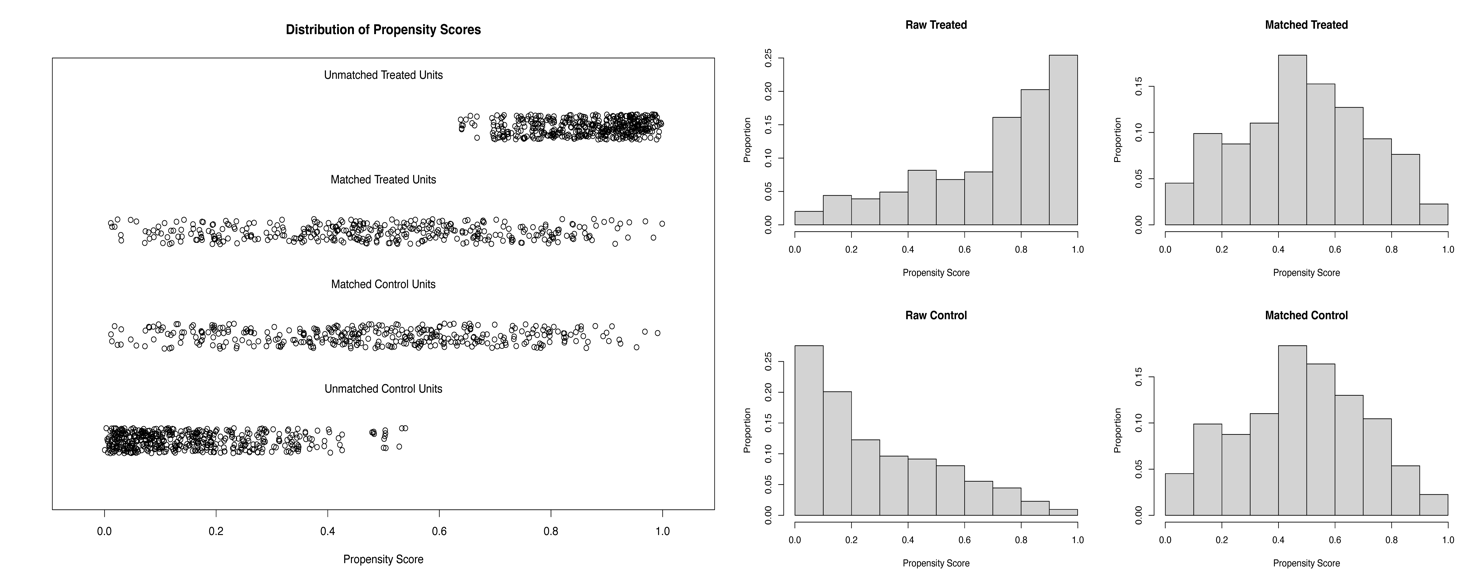


**Figure-S1**: Distribution of the propensity score in treated and control group, before and after matching.


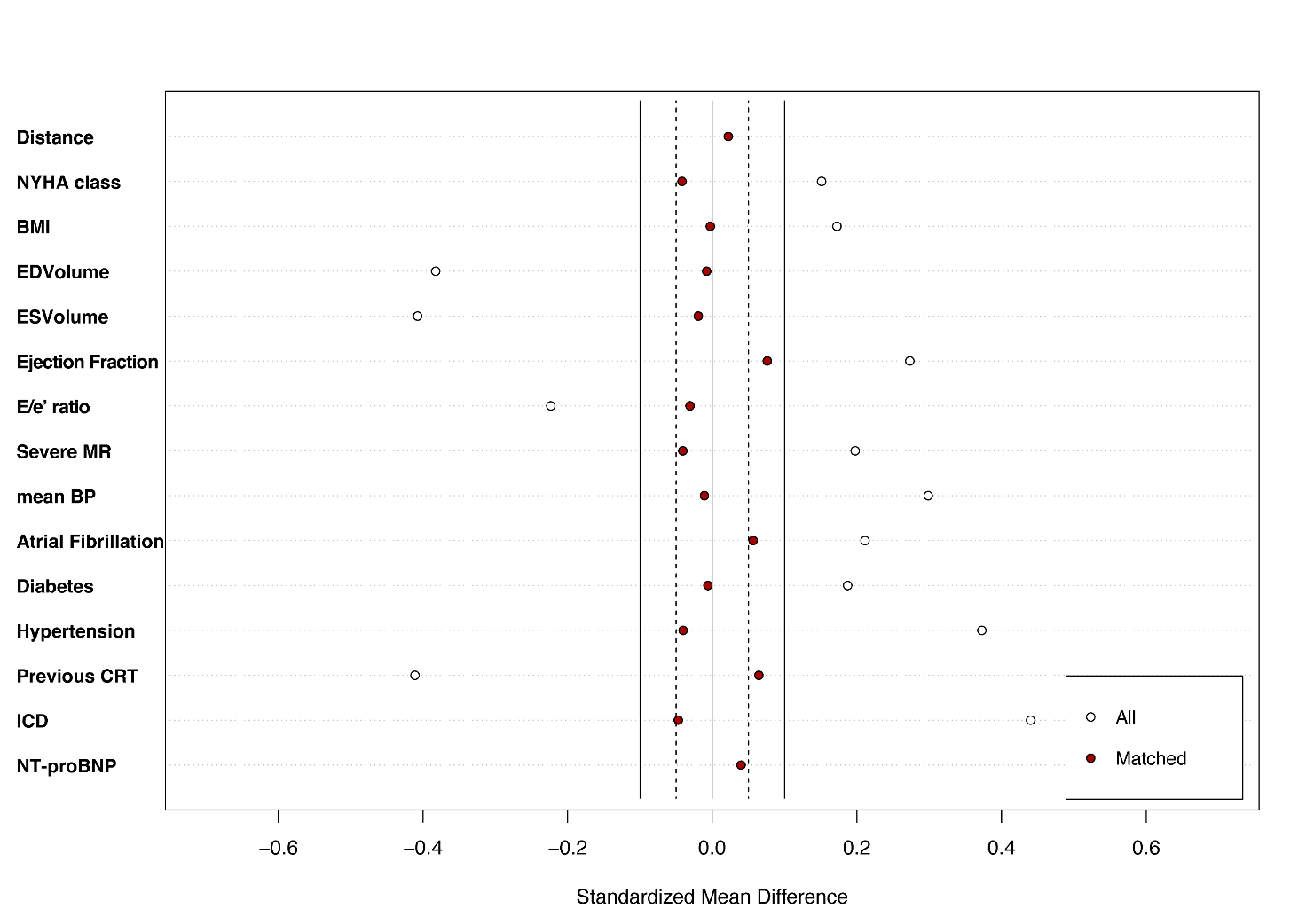


**Figure-S2**: Imbalance before and after matching for covariates in the propensity score model. BMI=Body mass index; EDVolume = end-diastolic volume; ESVolume = end-systolic volume; BP = blood pressure; CRT = cardiac resynchronization therapy; ICD = implantable cardiac defibrillator; NYHA = New York Heart Association; MR = mitral regurgitation.


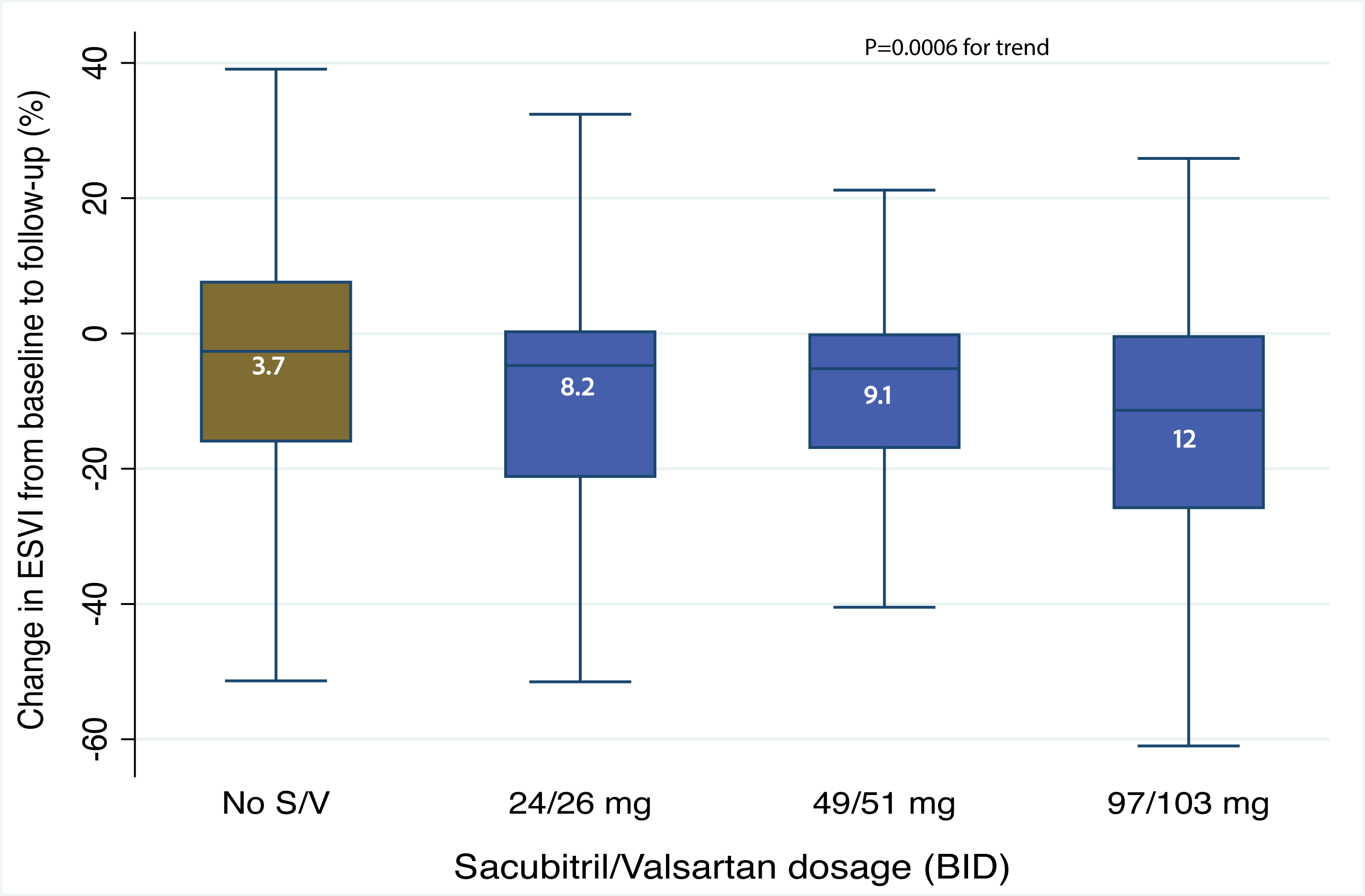


**Figure-S3**: End-systolic volume index (ESVI) changes from baseline to follow-up according to sacubitril/valsartan dosage.
